# From local to systemic: endoscopic findings reshape the diagnostic paradigm of satoyoshi syndrome – a case report

**DOI:** 10.1055/a-2851-8290

**Published:** 2026-05-21

**Authors:** Yiting Shi, Shiyun Mai, Huamei Ju, Xiaoqian Zhang, Huijing Zhang

**Affiliations:** 1 Department of Endoscopy 159407 The First Affiliated Hospital of China Medical UniversityShenyangChina; 2 Department of Neurology 159407 The First Affiliated Hospital of China Medical UniversityShenyangChina

Satoyoshi syndrome (SS) is a rare, multisystem disorder of presumed autoimmune etiology 1 2 3 , characterized by progressive muscle spasms, alopecia, and chronic diarrhea 4 . Gastrointestinal symptoms are core manifestations, but their endoscopic spectrum remains incompletely defined.

We report the case of a 50-year-old woman diagnosed through distinctive upper gastrointestinal findings during evaluation for abdominal discomfort. Endoscopy ( Fig. 1 and Video 1 ) revealed severe atrophic gastritis with multiple cystic polyps and two coral-like polypoid lesions in the mid-gastric body. The duodenum showed severe villous atrophy and polypoid lesions. Pathology ( Fig. 2 ) confirmed a low-grade gastric foveolar-type adenoma and a low-grade duodenal tubular adenoma. Contrast-enhanced computed tomography revealed localized, uneven edema and thickening of the gastric and duodenal walls with mucosal enhancement ( Fig. 3 ). This unusual combination of gastric cystic changes and severe duodenal villous atrophy suggested a systemic disease, prompting a comprehensive clinical evaluation.

**Fig. 1:**
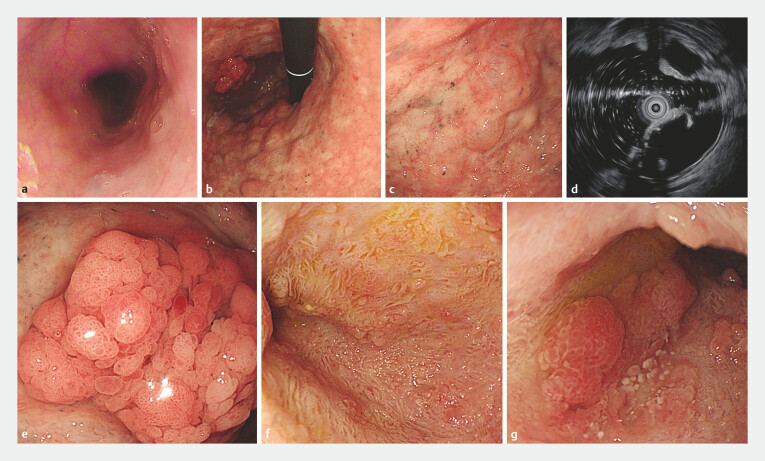
Upper gastrointestinal endoscopic findings in the patient. **a** No abnormalities were observed in the esophagus. **b** The gastric fundus and body mucosa showed diffuse severe atrophy with clearly visible vascular networks, exposure of submucosal vascular textures, flattening of mucosal folds, and numerous black dot-like appearances. **c** On the atrophic gastric body mucosa, multiple scattered cystic protrusions measuring approximately 0.3 to 0.8 cm in diameter were observed; they had smooth surfaces, were similar in color to the surrounding mucosa, and were soft to touch, and some appeared semi-transparent. **d** An EUS probe placed on one of the cystic protrusions in the gastric body showed that the lesion originated from the mucosal layer with a homogeneous anechoic area inside, and no posterior echo enhancement, confirming a cystic structure likely representing fundic gland cysts or mucosal cystic degeneration. **e** Two polypoid protrusions approximately 2.0 cm and 0.8 cm in size with a coral-like surface were seen on the greater curvature of the middle gastric body. **f** The bulb and descending parts of the duodenum showed severe mucosal atrophy; villous structures were almost completely lost, mucosal surfaces appeared flat and pale, and exhibited degenerative changes. **g** Polypoid protrusions are visible in some areas.

From local to systemic: Endoscopic findings reshape the diagnostic paradigm of Satoyoshi syndrome – a case report.Video 1

**Fig. 2:**
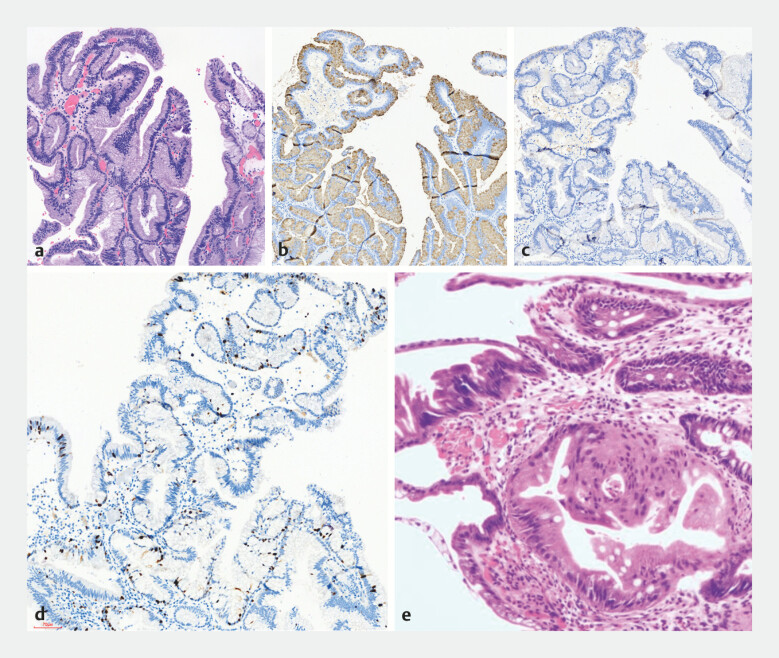
Pathological findings in the patient. **a** A biopsy was performed at the larger protrusion on the greater curvature of the gastric body, and histopathological examination showed that the tumor cells were arranged in papillary and tubular patterns, presented a high columnar shape with an abundant and clear cytoplasm, nuclei located at the basal part, and no obvious atypia, which was consistent with the diagnosis of gastric foveolar-type adenoma (low grade). **b** The results of immunohistochemical staining showed that the tumor cells diffusely expressed MUC5AC. **c** The cells did not express CDX2. **d** The Ki-67 proliferation index was approximately 1%. **e** a low-grade duodenal tubular adenoma.

**Fig. 3:**
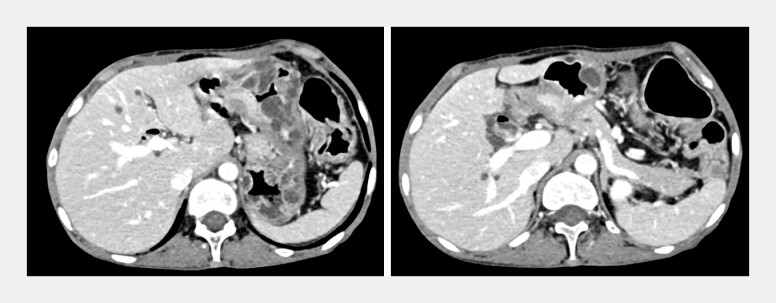
Contrast-enhanced CT reveals localized, relatively prominent, uneven edema and thickening of the gastric wall and duodenal wall, with mucosal enhancement observed during the contrast phase. CT, computed tomography.

History revealed typical features including short stature, generalized alopecia, paroxysmal muscle spasms, lower limb weakness, premature ovarian failure, and a butterfly-shaped rash ( Fig. 4 ). Laboratory tests showed mild anemia, hypoalbuminemia, positive autoimmune antibodies (AMA-M2, anti-nRNP/Sm, and anti-Sm), elevated gastrin-17, and a 22q11.21 microdeletion.

**Fig. 4:**
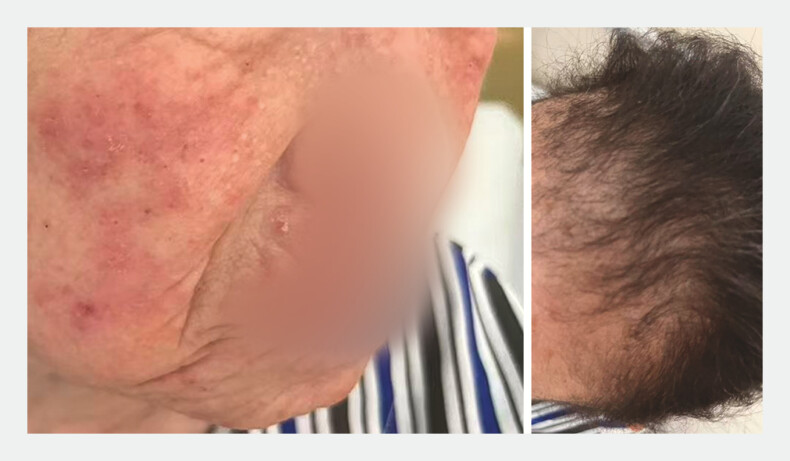
Physical examination of the patient reveals sparse hair, and a butterfly-shaped rash.

Final diagnosis was the SS, systemic lupus erythematosus (SLE), and gastric foveolar-type adenoma. Glucocorticoid treatment showed limited improvement, and the patient requested discharge.

This case highlights how unique endoscopic findings – gastric mucosal cystic changes and duodenal villous atrophy – led to the diagnosis of SS, enriching its endoscopic spectrum. This first report links the SS to gastric foveolar-type adenoma and 22q11.21 microdeletion, suggesting genetic susceptibility to multiple autoimmune diseases. The case underscores the endoscopist’s crucial role in integrating local findings with systemic clinical context to diagnose complex conditions. Treatment remains challenging, necessitating future targeted therapies.

We report a case of SS coexisting with SLE and gastric foveolar-type adenoma diagnosed through unique endoscopic findings, expanding the understanding of SS and emphasizing the endoscopist’s role.

Endoscopy_UCTN_Code_CCL_1AB_2AC_3AB

## References

[1] Satoyoshi E Yamada K Recurrent muscle spasms of central origin. A report of two cases Arch Neurol 1967 16 254 264 10.1001/archneur.1967.00470210030004 6018875

[2] Solís-García Del Pozo J de Cabo C Solera J Treatment of Satoyoshi syndrome: a systematic review Orphanet J Rare Dis 2019 14 146 10.1186/s13023-019-1120-7 31217029 PMC6585110

[3] Matsuura E Matsuyama W Sameshima T Satoyoshi syndrome has antibody against brain and gastrointestinal tissue Muscle Nerve 2007 36 400 403 10.1002/mus.20773 17405137

[4] Harris S Czarnecki K Naina HVK Chronic diarrhea, alopecia totalis and muscle cramps in a 36 year old female: 717 Off J Am Coll Gastroenterol | ACG 2007 102 S381 S382

